# Comparative Effectiveness of Behavioral Interventions on Quality of Life for Older Adults With Mild Cognitive Impairment: A Randomized Clinical Trial

**DOI:** 10.1001/jamanetworkopen.2019.3016

**Published:** 2019-05-17

**Authors:** Melanie J. Chandler, Dona E. Locke, Julia E. Crook, Julie A. Fields, Colleen T. Ball, Vaishali S. Phatak, Pamela M. Dean, Miranda Morris, Glenn E. Smith

**Affiliations:** 1 Division of Psychology, Mayo Clinic, Jacksonville, Florida; 2 Division of Psychology, Mayo Clinic, Scottsdale, Arizona; 3 Division of Biomedical Statistics and Informatics, Mayo Clinic, Jacksonville, Florida; 4 Division of Neurocognitive Disorders, Mayo Clinic, Rochester, Minnesota; 5 Department of Neurological Sciences, University of Nebraska Medical Center, Omaha; 6 Department of Psychiatry and Behavioral Sciences, University of Washington, Seattle; 7 Health Studies Unit, Mayo Clinic, Jacksonville, Florida; 8 Department of Clinical and Health Psychology, University of Florida, Gainesville

## Abstract

**Importance:**

Recommendations to engage in behavioral strategies to combat clinically significant cognitive and behavioral symptoms are routinely given to persons with mild cognitive impairment (MCI). The comparative effectiveness of these behavioral interventions is not well understood.

**Objective:**

To compare the incremental effects of combinations of 5 behavioral interventions on outcomes of highest importance to patients with MCI.

**Design, Setting, and Participants:**

In this multisite, cluster randomized, multicomponent comparative effectiveness trial, 272 patients from 4 academic medical outpatient centers (Mayo Clinic, Rochester, Minnesota; Mayo Clinic, Scottsdale, Arizona; Mayo Clinic, Jacksonville, Florida; and University of Washington, Seattle) were recruited from September 1, 2014, to August 31, 2016, with last follow-up March 31, 2019. All participants met the National Institute on Aging–Alzheimer’s Association criteria for MCI.

**Interventions:**

The intervention program was modeled on the Mayo Clinic Healthy Action to Benefit Independence and Thinking (HABIT) program, a 50-hour group intervention conducted during 2 weeks, including memory compensation training, computerized cognitive training, yoga, patient and partner support groups, and wellness education. In our study, 1 of 5 interventions was randomly selected to be withheld for each intervention group. Participants and their partners had 1-day booster sessions at 6 and 12 months after intervention.

**Main Outcomes and Measures:**

Quality-of-life measurement of participants with MCI at 12 months was the primary outcome, selected based on the preference rankings of previous program participants. Mood, self-efficacy, and memory-based activities of daily living were also highly ranked.

**Results:**

A total of 272 participants (mean [SD] age, 75 [8] years; 160 [58.8%] male and 112 [41.2%] female) were enrolled in this study, with 56 randomized to the no yoga group, 54 to no computerized cognitive training, 52 to no wellness, 53 to no support, and 57 to no memory support system. The greatest effect size for quality of life was between the no computerized cognitive training and no wellness education groups at 0.34 (95% CI, 0.05-0.64). In secondary analyses, wellness education had a greater effect on mood than computerized cognitive training (effect size, 0.53; 95% CI, 0.21-0.86), and yoga had a greater effect on memory-related activities of daily living than support groups (effect size, 0.43; 95% CI, 0.13-0.72).

**Conclusions and Relevance:**

These results provide further support for behavioral interventions for persons with MCI. Different outcomes were optimized by different combinations of interventions. These findings provide an initial exploration of the effect of behavioral interventions on patient-advocated outcomes in persons with MCI.

**Trial Registration:**

ClinicalTrials.gov identifier: NCT02265757

## Key Points

**Question:**

What is the comparative effectiveness of behavioral interventions for mild cognitive impairment on patient-selected outcomes of quality of life, mood, self-efficacy, and memory-related activities of daily living?

**Findings:**

In this cluster randomized comparative effectiveness trial of 272 randomized participants, withholding wellness education had a greater effect on mood than computerized cognitive training, and yoga had a greater effect on memory-related activities of daily living than support groups at 12 months after intervention. Computerized cognitive training had the least effect on these outcomes.

**Meaning:**

This study offers a starting point for understanding which behavioral interventions are most effective for patient- and partner-desired outcomes in mild cognitive impairment.

## Teaser

This cluster randomized, multicomponent comparative effectiveness trial compares the incremental effects of combinations of 5 behavioral interventions on outcomes of highest importance to patients with mild cognitive impairment.

## Introduction

The medical community is increasingly able to identify Alzheimer disease (AD) at an early stage, including the mild cognitive impairment (MCI) stage. Approximately 15% to 20% of people 65 years or older have MCI, and approximately one-third of these individuals develop dementia related to AD within 5 years.^1^ Amnestic MCI is defined as memory abnormality beyond normal age-related decline with relatively intact functional capacity or, in other words, not (yet) dementia.^2^

In the absence of US Food and Drug Administration–approved medical therapies for MCI, patients are often presented with numerous recommendations regarding nonpharmacologic interventions (eg, note taking, physical exercise, and computer memory games). There is promising evidence that such behavioral interventions can be beneficial in MCI. However, choosing among options can be overwhelming. A recent meta-analysis^3^ suggests that cognitive interventions have significant mild-to-moderate effect sizes (ESs) (Hedges *g*, 0.23-0.40) for patients with MCI on multiple cognitive domains, including memory, attention, and processing speed. A meta-analysis^4^ of noncognitive effects of cognitive interventions in patients with MCI demonstrated small but significant effects for activities of daily living (ADLs) (Cohen *d*, 0.23), mood (Cohen *d*, 0.16), and metacognitive outcomes (ie, how people think or feel about their memory) (Cohen *d*, 0.30). Physical exercise meta-analysis outcomes in individuals older than 50 years have provided similar moderate overall ESs on cognition (mean ES, 0.29).^5^

Only recently have enough studies been completed on individual interventions in cognitive rehabilitation, physical exercise training, or psychotherapy to allow meaningful meta-analyses such as these to emerge in the literature.^3,4,5^ Still, these reviews are challenged with making conclusions because of an array of various approaches or combinations to such interventions. There is a dearth of literature examining the effectiveness of various behavioral interventions compared with each other but an increase in support for the use of multicomponent interventions in dementia prevention efforts.^6^

Previous studies^7,8^ have compared outcomes for a compensatory cognitive rehabilitation intervention and a computerized cognitive training (CCT) intervention vs no treatment control groups in randomized clinical trials. In these studies, patient memory-related ADLs (mADLs) were significantly improved compared with controls in patients randomized to the cognitive rehabilitation intervention but not CCT. Sense of memory self-efficacy significantly improved for those trained in use of the memory support system (MSS) calendar but not in the CCT or control groups.^7,8^ Furthermore, partners in both treatment groups had stable mood and anxiety level, whereas partners in the control groups had worsening depression and anxiety level during 6 months.^9^ Similarly, a systematic review^4^ supported that various computerized interventions aimed at improving cognition may be associated with reduced anxiety and depression in patients with MCI, and persons with MCI trained with therapists in compensatory strategies, such as the MSS calendar training, have a greater benefit in ADLs, self-beliefs about memory, and confidence.

Most studies on behavioral interventions to date have focused on the effect on cognition for older adults or for those with MCI. However, the increasing culture of patient-centered care considers patients to be members of the health care team, with an active voice in their treatment. To our knowledge, no previous trials have used the input of patients with MCI or their family members in the study design. Thus, we established that patients with MCI and their partners rated patient quality of life (QOL), self-efficacy, mood, and mADLs to be the most important outcomes to target with behavioral interventions, which was the first aim of this larger study.^10,11^ In the present study, we sought to compare the pairwise incremental effectiveness of the 5 behavioral interventions of the Mayo Clinic Healthy Action to Benefit Independence and Thinking (HABIT) program, a 50-hour behavioral intervention program for people with MCI. We hypothesized that the lack of MSS or a support group would have the most effect on QOL and self-efficacy, lack of a support group or yoga would have the most effect on mood, and lack of MSS training would have the most effect on mADLs. Although CCT may have an effect on cognitive outcomes, we hypothesize that CCT would have minimal effect on patient- and partner-advocated outcomes in this study.

## Methods

Full details of the study protocol and recruitment have been reported previously^12^ and are summarized below. The trial protocol can be found in Supplement 1. Because this study was an intention-to-treat comparative effectiveness trial from the Patient Centered Outcomes Research Initiative, the study design was restricted to comparing evidence-based interventions with each other rather than with a control group. The conduct of the study was reviewed and approved by the institutional review boards at Mayo Clinic and the University of Washington. This study followed the Consolidated Standards of Reporting Trials (CONSORT) reporting guideline. All participants provided written informed consent, and all data were deidentified.

### Patient and Partner Engagement

Before finalizing the trial design, we created stakeholder and patient and partner advisory groups. The patient and partner advisory group encouraged the conduct of the study to ensure that all participants received substantial treatment. They specifically favored and endorsed a study design that randomly suppressed only 1 of the 5 components of HABIT over a design that involved patients with MCI receiving 1 or none of the interventions. Thus, we adopted a fractional factorial design for comparative analysis,^13,14^ examining the cost of withholding rather than the benefit of adding an intervention.

### Participants

We planned to recruit 300 dyads that consisted of a patient with MCI and a partner, anticipating an approximate 10% attrition rate based on prior research.^15^ Participants were recruited through clinical services at the Mayo Clinic on the Rochester, Minnesota, Scottsdale, Arizona, and Jacksonville, Florida, campuses as well as at the University of Washington in Seattle from September 1, 2014, to August 31, 2016, with the last follow-up on March 31, 2018 and the last day of analysis on March 6, 2019. Consecutive candidates with a diagnosis of amnestic MCI (single or multidomain)^2^ were screened as potential study participants. Inclusion criteria comprised a Clinical Dementia Rating^16^ score of 0.5 or lower, not taking or stable while taking nootropic medication for 3 months or more, fluency in English, and attendance with a cognitively normal (Mini-Mental Status Examination^17^ score >24) care partner with at least twice-weekly contact with the patient with MCI. Exclusion criteria included current participation in another treatment-related clinical trial or major auditory and visual or motor impairment that affected ability to participate in the program. Patients with MCI completed the Dementia Rating Scale-2^16^ as a measure of general cognitive function at baseline.

### Intervention and Randomization

The 4-component interventions were delivered for 40 hours during 2 weeks. Participants were enrolled by study coordinators before randomization. This was a cluster randomized trial in which the unit of randomization was group session. Specifically, randomization was performed by a statistician (J.E.C.); 5 groups of patients and their partners at each site were randomly assigned to 1 of the 5 arms (each consisting of 4 components of HABIT), with the blocking constraint that each site had at least 1 (but no more than 2) groups assigned to each of the arms. Each assignment was concealed by the statistician for as long as possible; nonrecruiting study team members had assignments revealed to them on a need-to-know basis for scheduling purposes, but assignments were concealed from all others. The randomization assignment of a group was concealed from participants until they arrived for their first day of the program. Individuals collecting outcome measures were masked to group randomization. Each group session consisted of up to 20 dyads randomized to receive the same 4 of 5 HABIT components. Resulting randomization and allocation to each group per site are given in Table 1.

**Table 1.** Participant Baseline Characteristics by Study Arm

Characteristic	No Yoga (n = 56)	No CCT (n = 54)	No Wellness Education (n = 52)	No Support Groups (n = 53)	No MSS (n = 57)
Age, mean (SD), y	74.3 (7.3)	75.8 (8.0)	76.7 (7.3)	75.1 (7.3)	74.1 (7.9)
Education duration, median (range), y	17 (9-20)	16 (12-20)^a^	16 (12-20)	16 (10-20)	16 (6-20)
Male, No. (%)	34 (60.7)	33 (61.1)	30 (57.7)	32 (60.4)	31 (54.4)
Race other than white, No. (%)^b^	3 (5.4)	1 (1.9)	1 (1.9)	2 (3.8)	5 (8.8)
Partner relationship, No. (%)					
Spouse or partner	52 (92.9)	44 (81.5)	42/50 (84.0)	48 (90.6)	43/55 (78.2)
Son or daughter	2 (3.6)	6 (11.5)	4/50 (8.0)	3 (5.7)	8/55 (14.5)
Using nootropics, No. (%)	27/55 (49.1)	24 (44.4)	16/49 (32.7)	11 (20.8)	22/53 (41.5)
DRS-2 total score, mean (SD)	130.1 (8.7)^c^	127.5 (9.3)	128.0 (8.0)^a^	130.9 (7.6)	129.9 (8.8)^d^
CDR score, No. (%)					
0, None	4/55 (7.3)	1 (1.9)	5 (9.6)	4 (7.5)	7 (12.3)
0.5, Questionable	51/55 (92.7)	53 (98.1)	47 (90.4)	49 (92.4)	50 (87.7)
REACH anxiety score, mean (SD)	17.9 (5.4)	16.9 (5.4)^d^	18.1 (5.2)^e^	17.6 (5.6)^a^	17.8 (4.8)^f^
QOL-AD score, mean (SD)	39.9 (5.9)	40.9 (5.7)^a^	39.1 (5.0)^e^	41.5 (5.7)	39.9 (5.5)
Mood score, mean (SD)	11.5 (8.2)^1^	12.4 (9.6)^a^	12.6 (8.5)^e^	10.5 (7.2)	12.8 (6.8)^f^
Self-efficacy score, mean (SD)	74.1 (13.0)	74.1 (15.7)^a^	73.0 (12.1)^e^	75.6 (13.8)^a^	72.6 (15.2)^f^
mADL score, mean (SD)	18.2 (4.9)	19.2 (5.4)	18.3 (4.9)^f^	18.9 (4.8)^a^	18.9 (4.7)^f^

Abbreviations: CCT, computerized cognitive training; CDR, Clinical Dementia Rating; DRS-2, Dementia Rating Scale-2; mADL, memory-related activities of daily living; MSS, memory support system; QOL-AD, Quality of Life in Alzheimer Disease; REACH, Resources for Enhancing Alzheimer Caregiver Health.

^a^ Data missing for 1 patient.

^b^ Participants self-selected race from white, Hispanic or Latino, black/African American, Asian/Pacific Islander, American Indian/Alaskan Native, or other.

^c^ Data missing for 7 patients

^d^ Data missing for 2 patients.

^e^ Data missing for 3 patients

^f^ Data missing for 4 patients.

Each of these components was originally chosen on a theoretical basis because each had support individually in the literature for effectiveness compared with no-treatment controls across a variety of outcomes (eg, cognitive functioning, QOL, mood, and partner burden).^4,18^

Components of HABIT include 45- to 60-minute sessions of the following: yoga, CCT, wellness education, support groups, and MSS. Yoga was a combination of physical exercise and relaxation training with certified yoga instructors. A customized DVD was provided to encourage continued practice after the intervention.^19^ For CCT, instruction was provided in the use of a commercially available BrainHQ product from Posit Science Corporation.^20^ A 1-year subscription was provided to encourage continued use after the intervention. Wellness education consisted of lectures that covered a range of health topics, including living with MCI, sleep hygiene, depression, nutrition, and assistive technology. Dyads were given resources and written information to help incorporate healthy behavioral changes discussed in the lectures (eg, improving diet) into their lives after the intervention. For the support group intervention, patients with MCI and their partners met separately in therapist-led support groups. The patients with MCI group focused on reminiscence and discussion of MCI-related concerns. The partner group was a traditional support group focused on caregiving themes. For MSS, patients with MCI received cognitive rehabilitation daily, which focused on a compensatory-focused calendar and note taking.^8,21^ This intervention involved training from a structured curriculum in the use of the MSS to develop compensatory written reminders for important appointments, tasks, or experiences and thoughts of the day. Patients with MCI and their partners were provided the MSS in an ongoing manner to enable continued use after the intervention.

For this trial, care was taken to exclude presentation of material related to the suppressed component within another component to avoid overlap or treatment diffusion. For example, all wellness education references to exercise were excluded if the yoga component was excluded.

### Outcome Measures

The QOL of the patients with MCI at 12-month follow-up was the primary outcome measure as assessed by the QOL-AD overall score.^22^ Mood was measured by the Center for Epidemiologic Studies Depression Scale,^23^ and self-efficacy was assessed using modified, selected items from the Chronic Disease Self-efficacy Scales.^24^ Patient functional memory was assessed by 8 mADLs on the informant-based Everyday Cognition questionnaire.^25^ All measures were completed at baseline, treatment end, 6 months after intervention, and 12 months after intervention. Six- and 12-month data collection was part of a 1-day booster session that included a refresher for each of the 4 components originally provided (described further elsewhere^12^). All outcome data were collected before the commencement of the booster session.

### Statistical Analysis

For the primary analysis, we used a longitudinal mixed-effects regression model to compare QOL at 12 months among the 5 groups. The analysis used data from all 4 time points (baseline, end of treatment, 6 months, and 12 months). Specifically, the model was such that QOL at baseline was modeled with fixed effects for age, sex, and site. The mean change in QOL from baseline to each of the 3 follow-up time points was modeled with fixed effects for group, age, and sex. In addition to the variation arising summarized by the usual residual error and to adjust for dependencies, we included random effects for patient group session because this was the unit of randomization (and to account for commonalities), for time nested within group (to account for time-specific commonalities within patient groups), and for participant (to account for person-specific dependence in the multiple measures over time).

The pairwise differences in group effects on QOL at 12 months were of primary interest, with the same pairwise differences in effects of mood, self-efficacy, and mADLs at 12 months of secondary interest. In addition, we sought to examine the fitted trajectories over time for each of the 5 groups for each of the 4 outcome measures. We constructed 95% CIs using the profile likelihood method and performed testing using corresponding likelihood ratio tests. We assessed the significance of each of the 10 pairwise comparisons of each outcome accounting for multiple testing with Westfall stepwise adjustment. Two-sided adjusted *P* < .05 was considered to be statistically significant. The ES for a given measure at 12 months was calculated as the fitted mean difference between study arms at 12 months and dividing it by the baseline SD of QOL measures. Analyses were performed using R statistical software, version 3.2.3 (R Foundation for Statistical Computing).

Data from a prior patient sample^26^ of participants in the full 5-component HABIT and an untreated control group from a prior randomized clinical trial^8^ were used to inform statistical power. Estimated SEs were extracted from the fitted models from which we could estimate SEs for different sample size scenarios. We originally determined the study with 300 participants, and 10% attrition would be powered (80% at 5% significance level) to detect an effect of any 1 of the 5 interventions on QOL at 12 months of 0.53 baseline SDs. However, subsequent analysis proved this to be an underestimate (see the Discussion section).

## Results

### Participants

**Figure 1.**
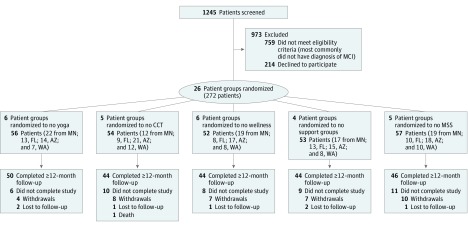
Recruitment CONSORT Chart AZ indicates Arizona; CCT, computerized cognitive training; FL, Florida; MCI, mild cognitive impairment; MN, Minnesota; MSS, memory support system; and WA, Washington.

A total of 272 participants (mean [SD] age, 75 [8] years; 160 [58.8%] male and 112 [41.2%] female) were enrolled in this study (Figure 1). No meaningful differences were found in demographics or general cognitive status across sites or groups at baseline (Table 1). There were slightly more participants in the no support group arm who were receiving memory medications, although subsequent analyses found no meaningful effect on outcomes of use of memory medication across groups (eFigure in Supplement 2). A total of 228 participants (83.8%) completed at least 12 months of follow-up. Of the 44 (16.2%) who did not, 1 (2.3%) died, 36 (81.8%) formally withdrew (most common reasons: 11 [30.6%] because of health concerns of participant or partner, 4 [11.1%] because of insurance or billing issues, and 3 [8.3%] because of changes in living situation attributable to moving or divorce), and 7 (15.9%) were unavailable for follow-up. No meaningful differences were observed between 12-month follow-up completers and noncompleters (eTable 1 in Supplement 2) other than completers tended to more often have a spouse for a partner (88%) compared with noncompleters (75%). No adverse effects were detected as a result of this study.

### Outcome Measures

After multiple testing was adjusted for, no statistically significant evidence was found of pairwise differences in incremental effectiveness of interventions on the primary outcome (QOL) at 12 months (Table 2). The largest ES indicated that wellness education was more likely to have an effect than CCT (ES, 0.34; 95% CI, 0.05-0.64; *P* = .15).

**Table 2.** Comparative Incremental Effects on QOL at 12 Months for Pairs of HABIT Components^a^

HABIT Components in Common	Components Compared	Difference in QOL at 12 mo (95% CI)	*P* Value	
			Original	Adjusted
Yoga, SG, and MSS	WE-CCT	0.34 (0.05 to 0.64)	.02	.15
CCT, SG, and MSS	WE-yoga	0.26 (−0.03 to 0.55)	.08	.37
Yoga, CCT, and MSS	WE-SG	0.22 (−0.08 to 0.52)	.15	.56
Yoga, WE, and SG	MSS-CCT	0.19 (−0.10 to 0.49)	.20	.63
Yoga, CCT, and SG	WE-MSS	0.15 (−0.14 to 0.45)	.31	.75
Yoga, WE, and MSS	SG-CCT	0.13 (−0.17 to 0.42)	.40	.82
CCT, WE, and SG	MSS-yoga	0.11 (−0.18 to 0.40)	.45	.83
WE, SG, and MSS	Yoga-CCT	0.08 (−0.20 to 0.37)	.57	.84
Yoga, CCT, and WE	MSS-SG	0.07 (−0.23 to 0.37)	.65	.86
CCT, WE, and MSS	SG-yoga	0.04 (−0.25 to 0.33)	.76	.86

Abbreviations: CCT, computerized cognitive training; HABIT, Healthy Action to Benefit Independence and Thinking; MSS, memory support system; QOL, quality of life; SG, support group; WE, wellness education.

^a^ To ease interpretation, the 2 components compared are ordered such that the first component listed has a higher estimated mean QOL at 12 months than the second component. Differences in QOL are shown in units of the SD of baseline QOL. Both original and multiple-test adjusted *P* values are shown.

**Figure 2.**
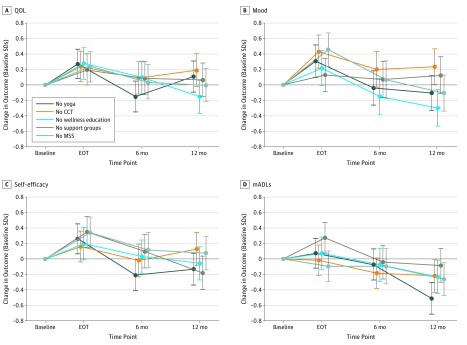
Effect Sizes by Study Arm and Intervention Effect sizes were estimated from linear mixed-effects regression models, in which a 1-unit increase in the effect size corresponded to a 1-SD improvement in patient outcome. Baseline SDs were 5.59 for quality of life (QOL) (A), 4.95 for memory-related activities of daily living (mADLs) (D), 8.11 for mood (B), and 14.00 for self-efficacy (C). Treatment occurred for 10 days over a 2-week period. CCT indicates computerized cognitive training; EOT, end of treatment; and MSS, memory support system. Error bars represent 95% CIs for the effect sizes.

In a secondary analysis of mood at 12 months (Table 3), the largest ES was again between wellness education and CCT, this time with statistical significance (ES, 0.53; 95% CI, 0.21-0.86; *P* = .01). No statistically significant differences were found between groups on self-efficacy after multiple comparison adjustment. For mADLs, yoga was more effective than support groups (ES, 0.43; 95% CI, 0.13-0.72; *P* = .04). Although not significant after adjustment for multiple analyses, MSS (ES, 0.34; 95% CI, 0.02-0.66; unadjusted *P* = .04) and yoga (ES, 0.34; 95% CI, 0.02-0.66; unadjusted *P* = .04) were potentially more effective than CCT, and wellness education was also more effective than support group (ES, 0.42; 95% CI, 0.09-0.75; unadjusted *P* = .01). For self-efficacy, support group was estimated to be more effective than CCT (ES, 0.31; 95% CI, 0.01-0.61; unadjusted *P* = .04). Figure 2 shows trajectories over time for each study arm and each of the 4 outcomes. Outcomes by site are presented in eTable 2 and eTable 3 in Supplement 2.

**Table 3.** Comparative Incremental Effects on Secondary Outcomes at 12 Months for Pairs of HABIT Components When Added to the Remaining 3 Components of HABIT^a^

Comparison	Effect Size (95% CI)	*P* Value
**Mood**		
WE-CCT	0.53 (0.21 to 0.86)	.01
WE-SG	0.42 (0.09 to 0.75)	.08
MSS-CCT	0.34 (0.02 to 0.66)	.19
Yoga-CCT	0.34 (0.02 to 0.66)	.19
Yoga-SG	0.23 (−0.09 to 0.55)	.53
MSS-SG	0.23 (−0.10 to 0.56)	.53
WE-Yoga	0.19 (−0.13 to 0.51)	.60
WE-MSS	0.19 (−0.13 to 0.52)	.60
SG-CCT	0.11 (−0.22 to 0.44)	.76
MSS-yoga	0.00 (−0.32 to 0.32)	>.99
**Self-efficacy**		
SG-CCT	0.31 (0.01 to 0.61)	.26
Yoga-CCT	0.26 (−0.03 to 0.55)	.39
SG-MSS	0.26 (−0.05 to 0.56)	.42
Yoga-MSS	0.21 (−0.08 to 0.50)	.57
WE-CCT	0.18 (−0.12 to 0.48)	.67
WE-MSS	0.13 (−0.17 to 0.43)	.85
SG-WE	0.12 (−0.18 to 0.43)	.85
Yoga-WE	0.08 (−0.22 to 0.37)	.93
MSS-CCT	0.05 (−0.25 to 0.35)	.93
SG-Yoga	0.05 (−0.25 to 0.34)	.93
**mADLs**		
Yoga-SG	0.43 (0.13 to 0.72)	.04
Yoga-CCT	0.29 (0.00 to 0.58)	.27
Yoga-WE	0.28 (−0.02 to 0.57)	.31
Yoga-MSS	0.25 (−0.04 to 0.54)	.38
MSS-SG	0.18 (−0.12 to 0.48)	.69
WE-SG	0.15 (−0.16 to 0.45)	.79
CCT-SG	0.14 (−0.16 to 0.44)	.79
MSS-CCT	0.04 (−0.26 to 0.34)	.99
MSS-WE	0.03 (−0.27 to 0.33)	.99
WE-CCT	0.01 (−0.29 to 0.31)	.99

Abbreviations: CCT, computerized cognitive training; HABIT, Healthy Action to Benefit Independence and Thinking; mADLs, memory-related activities of daily living; MSS, memory support system; SG, support group; WE, wellness education.

^a^ To ease interpretation, the 2 components in any comparison are ordered such that the first component listed has higher outcome at 12 months than the second component. *P* values are multiple-test adjusted within each outcome.

## Discussion

In 2017, the National Academies of Sciences, Engineering, and Medicine concluded that although data do not currently support a widespread campaign for any particular strategy aimed at delaying or preventing cognitive decline in aging and MCI, there are encouraging results for blood pressure management and 2 behavioral interventions: cognitive training and physical activity.^27^ In this study, we sought to examine the comparative effectiveness of behavioral interventions for patients with MCI, including cognitive training (MSS or CCT) and physical activity (yoga), as well as the effect of wellness education and supportive group therapy.

This was a patient-centered, comparative effectiveness study. As such, the primary and secondary outcomes were determined by former patients as QOL (primary), self-efficacy, mood, and mADLs.^11^ Furthermore, we were prompted to forgo the traditional additive approach of randomly adding participants to 1 intervention at a time to undertake a novel, subtractive approach of randomly withholding 1 intervention at a time.^13,14^ This approach ensured that all participants would receive substantial interventions while still permitting comparison of each component.

We attained a high (84%) retention rate, in keeping with a previous recruitment and retention trial in a different sample of 86% (range, 74%-94%).^15^ In all groups, most outcomes were improved by end of treatment, although by 12 months most outcomes were no longer significant. These within-group trajectories must be interpreted cautiously because there was no untreated control group in this study. Still, spontaneous improvement in QOL, mood, self-efficacy, and mADLs have not been observed in previous work with no-treatment controls^7^ and is not the expected natural trajectory in MCI, which tends toward deterioration in QOL, mood, and function.

We acknowledge that in this study interpretation of the effect of not receiving an intervention component countered the design of most clinical trials. In an attempt to simplify the explanation of the results, we note that pairwise comparisons of groups are interpretable as comparisons of the incremental effects of one intervention to another when all patients received the remaining 3 interventions. There was evidence of differences in mood and mADLs among groups at 12 months. Namely, those who had wellness education had significantly better mood at 12 months than those who had CCT. Although we assume that this means that wellness education was more effective, we cannot rule out the possibility that participating in CCT may actually have been detrimental to long-term mood. Furthermore, yoga was significantly more important to mADLs than was a support group. Although not significant after adjustments for multiple comparisons, moderate ESs were obtained that suggest that wellness education was most important to QOL at 1 year after the intervention, yoga and MSS were also valuable for mood outcomes, and support groups most positively affected self-efficacy.

These findings were only partially supportive of our hypotheses. Of note, we underestimated the relative effect of wellness education on mood and perhaps on QOL. Yoga may have been effective for mood as hypothesized but also was significantly effective for mADLs. Although the evidence is less clear, support groups may have been more effective compared with the other interventions on self-efficacy (but not QOL), and MSS may have been more effective for mood than it was for the other outcomes for which it was expected to be more effective. We accurately hypothesized that CCT would have little effect on the outcomes of interest, although it may have positive effects (eg, on actual cognition).

Thus, for participants who shared the same opinions as our HABIT alumni in prioritizing patient QOL, self-efficacy, mood, and functional ability,^11^ participation in a program that included wellness education, yoga, MSS calendar training, and a support group provided the most benefit in the long term. Computerized cognitive training appeared to be of little to no benefit compared with the other interventions with respect to these outcomes. Of note, when asked to rank the importance of the interventions they received, the same alumni group listed (from most to least important) (1) MSS, (2) support group, (3) CCT, and (4 and 5 [tied]) yoga and wellness education.^11^ This finding supports the perceived importance of MSS and support groups but raises questions about whether the usefulness of CCT may be better reflected by cognitive outcome measures and whether patient and partners appreciate the potential benefits of yoga or wellness education. To our knowledge, this was the first large-scale trial of behavioral interventions tailored to the outcomes of greatest importance to patients with MCI and their support network. Other outcomes, including cognition, time to dementia diagnosis, partner QOL, mood, and burden, were collected as secondary outcomes in this study and are still under investigation.

### Limitations

Our study had a novel statistical design, and our initial statistical power assessment was unrealistic. We subsequently have determined that for pairwise comparisons, we were powered (80% power at the 5% significance level) to only detect differences between groups as high as 0.72 baseline SDs or even higher, taking into account multiple testing adjustment. Thus, the study was not sufficiently powered. However, the results still provide preliminary answers regarding comparative effectiveness. Given this finding, we thought it important to highlight pairwise comparisons with moderate ESs that were significant before multiple analysis adjustments because a future, better powered study may find those results to be significant.

Although we used our own version of these interventions, similar behavioral interventions (eg, therapy-based cognitive rehabilitation, CCT programs, physical exercise, psychotherapy or group therapy, and education programs for memory loss) are being offered in various combinations in medical and research centers around the world.^4^ Although our exact conclusions may not be generalizable to other forms of behavioral intervention (eg, resistance training as physical exercise vs yoga), we offer these results to encourage further comparative effectiveness trials. Future research can look at the nuances of different types of physical exercise compared with different types of therapist-based cognitive rehabilitation and combinations thereof. Further limiting potential generalizability of the results, the recruited cohort was ultimately not sufficiently diverse and was highly educated. In addition, all outcomes of interest presented were subjective reports. We did not include any biomarkers, such as cortisol levels, to measure mood and stress or objective performance-based mADLs that could have added strength to the findings.

When determining the most important outcomes to patients and partners, we surveyed HABIT participant in the 6 years before our study.^11^ However, most responders had recently completed the program, on average just more than a year since diagnosis. Thus, the results are most applicable to those relatively early in the MCI diagnosis. Still, this is likely the best time for the types of behavioral interventions included in HABIT. Asking participants earlier (before MCI) or later (dementia) may have led to preference of different outcomes.

## Conclusions

Although there were no statistically significant differences, it appears that wellness education may be the most effective of the 5 HABIT program components on QOL 1 year after intervention, whereas CCT may be the least effective. Wellness education was significantly more effective than CCT for mood, and yoga was more effective for mADLs than a support group.

## Notes

Supplement 1. **Trial Protocol**

Supplement 2. **eFigure.** Mean Changes in Quality of Life **eTable 1.** Baseline Characteristics According to Length of Follow-up **eTable 2.** Outcome Measures at Baseline by Study Group and Site **eTable 3.** Summaries of Raw Changes in Outcome Measures from Baseline to 12 Months by Study Group and Site

Supplement 3. **Data Sharing Statement**
