# Supplementary material for: Comparative Effectiveness of Behavioral Interventions on Quality of Life for Older Adults With Mild Cognitive Impairment: A Randomized Clinical Trial
Source: JAMA Netw Open. 2019 May 17;2(5):e193016. doi: 10.1001/jamanetworkopen.2019.3016 (PMC6537922; doi:10.1001/jamanetworkopen.2019.3016)
Supplement: Supplement 1. — Trial Protocol [file jamanetwopen-2-e193016-s001.pdf]

Aims

1) To engage consumers/patients and their care partners in the
   a. prioritization of outcomes for persons diagnosed with mild cognitive impairment
   b. evaluation of likely contributions of different behavioral interventions to those outcomes.
2) To incorporate the results of Aim 1into a comparative effectiveness study involving 5 components of a multicomponent behavioral intervention intended to prevent or delay the development of dementia in persons with mild cognitive impairment.
3) To demonstrate the efficiency of our novel 'subtraction design' and statistical analysis methods for the evaluation of multicomponent interventions (relative to traditional trial designs).

Methods:

Aim 1. In aim 1 of the study, Dr. Smith will oversee refinement, extension, and validation of this process. Initially, we will conduct focus groups with our Participant (aka Patient) and Partner Advisory Group (PPAG) and our stakeholder advisory group (SAG; See Appendix A) to refine our interviews and surveys. To guide this effort we will consult Dr. Ashok Kumbamu, a qualitative researcher funded through the Biostatistics, Epidemiology, and Research Design Resource of the Mayo Clinical and Translational Science Award (CTSA) to support qualitative research within Mayo.

Focus Groups. Our approach to conducting focus groups will ensure quick and quality data collection by recruiting using our motivated PPAG members, developing strong protocols, using experienced moderators, and rapidly analyzing results. Within 6 weeks of award, we will have conducted and summarized our focus groups. We plan to conduct three focus groups: one for patients, one for care partners and one for stakeholders. For the convenience our couples, we will likely hold the patient and care partner groups consecutively on the same day. The standard telecommunications system we use to conduct HABIT staff meetings across the sites will be used to allow us to direct focus groups across all sites from Rochester. Our PPAG has been select to assure diversity of age, gender, MCI etiology (e.g. probable Alzheimer, vs. Lewy Body dementia), time since diagnosis, care partner type, socioeconomic background and geography.

Development of Focus Group Protocols. The draft protocols will move from general perspectives and experiences related to mild cognitive impairment, patient-centered care, shared decision making, and understanding of treatment alternatives, to preferences for survey methodology (mail, phone, Internet, IVR) and reporting and finally to discussions of outcome priorities.

Data recording and Analysis. All focus groups will be audiotaped and attended by a note-taker. Topline notes will be obtained for each focus group. In consultation with Dr. Kumbamu, we will use a variety of well-established techniques, including data reduction, generation of themes, and validation of themes and findings to draw conclusions from the qualitative data.

Survey Refinement. Using the outcomes of the focus groups, Dr. Smith will refine our current survey regarding outcome priorities and intervention contributions to those outcomes.

Implementation of the Survey. Dr. Smith will direct the Mayo Clinic Survey Research Center (SRC) to coordinate and administer the refined survey. The target of this survey will be the remainder of the approximately 200 couples that will have participated in in the clinical HABIT program by the start of this project. For these couples, 46% of the people with MCI are women, the mean age of the group is 73.9, and the mean DRS-2 score is 127.7. Spouses make up 85% of care partners, adult children account for another 9%. The mean age of care partners is 69.3 years. The HABIT program maintains a centralized database of all of these program completers. We will provide the list of HABIT alumni to the Mayo Clinic SRC. Participants will be surveyed by mail with a target of 80% completions for care partners and 60% completions by patients (owing to the likely progress of cognitive impairments in HABIT participants who completed the program in the early years). The mail administration of the survey will follow four steps. Two weeks prior to the first mailing, prospective respondents will be sent a pre-notification letter to detect unknown changes of address and improve

response rates. Questionnaires will be sent 2 weeks after the initial contact letter along with a postage-paid return envelope. Patients who have not responded after 2 weeks will receive a second mailing. Individuals who have not responded after an additional 1 week will be contacted by telephone. Each telephone number in the non-responding sample will be called at various times of day and on different days of the week until there are 10 “no answer” dispositions. Another 10 attempts to interview will be made once a contact is made. Respondents successfully contacted will be encouraged to mail in their completed questionnaires or to complete a telephone interview. If we are told the selected respondent no longer lives in the household, we will ask for a new address and/or telephone number. Because of our on-going contact with HABIT alumni we will be able to complete survey analyses within the first 3 months of the grant. The survey is included in Appendix A.

Aim 2

Description of Subjects. Potential intervention participants will be identified from referrals to the HABIT program that come from the Neuropsychology and Behavioral Neurology clinics and/or Alzheimer’s Disease Research Center clinical cores of each participating site. All subjects will have a consensus diagnosis of amnestic MCI (either single domain or multi-domain). MCI diagnosis will be based on NIA-Alzheimer Association criteria.[1] Medical history, symptom profile, physical exam, and neuropsychological testing are reviewed by the neurologists and neuropsychologists in and a consensus diagnosis made via National Alzheimer Coordinating Center guidelines. All patients with a diagnosis of amnestic forms of MCI are provided a recommendation to participate in the clinical HABIT program. Consecutive HABIT candidates with consensus diagnoses of amnestic MCI (single domain or multi-domain) and meeting the inclusion/exclusion criteria will be approached for consent.

Inclusion Criteria:

a. A candidate for the HABIT program with a diagnosis of amnestic MCI (single domain or multi-domain).
b. .
c. Clinical Dementia Rating scale score <=0.5.
d. A cognitively normal care partner screened with the Mini Mental State Exam (>24) who has at least twice-weekly contact with the participant.
e. Either not taking or stable on nootropic(s) for at least 3 months.
f. Fluent in English. (Expanding the program to communities of Spanish-speakers will be a high priority in subsequent dissemination work).

Exclusion Criteria:

g. Inclusion in another clinical trial that would exclude participation. Subject will be considered for participation at the end of such a trial or as appropriate.
h. Medically unable to participate in all arms by virtue of visual or auditory impairments or non-ambulatory status.

These proposed inclusion/exclusion criteria will be vetted with our PPAG and SAG groups and affirmed or modified before initiation of the study.

By sharing staff, each site will have the capacity to see 100 HABIT participants per *16 month period (5 sessions with 20 participants each)*. This frequency will *permit us to enroll 300* potential participants over the *16 month* period targeted for enrollment. Based on our prior research we anticipate only a 10% attrition rate, leaving *270* complete datasets.

Randomization

Subtraction not addition. Traditional randomized controlled trials (RCTs) can be thought of as ‘additive’ trials where randomization leads to the addition of treatments beyond placebo. Participants are confronted with the probability of

receiving placebo (no treatment). This leads many potential participants to not consent or to withdraw if they believe they are receiving no treatment. In contrast, the proposed trial will be 'subtractive'. Data analysis will focus on which groups have the weakest outcomes as a result of missing a given intervention (see data analysis below). This innovative approach to randomization will involve suppression of just 1 of the 5 treatment components. Thus, all participants will receive at least 80% of the menu of interventions offered in this trial.

Sessions not people: Individualized randomization poses risk for diffusion of treatment effects, as the group nature of HABIT permits participants to compare their experiences. There is no blinding of intervention. Thus, we will randomize by session. HABIT is offered four times per year at each of 4 sites. Hence, in *16-months* there will be the opportunity to randomize *20*sessions (*5each at the 4 sites)*. We will employ block randomization, assuring that randomization to each of the 5 arms of the study results in at *55* participants per arm and that all sites run each arm once. All randomization will be overseen by Dr. Crook (the statistician) and handled by the data management center in Rochester.

Interventions

The HABIT program consists of 10-days of intervention over two weeks. Though the participants are given the weekend off, they are given 'homework' to practice each trained component on their own. With one of the 5 components randomly suppressed in the current design, each participant and care partner will receive 4 hours (4 components by 1 hour each) of intervention daily. As noted above HABIT programming initiates new healthy behavioral habits that MCI patients sustain with the support of care partners cuing.

Booster Sessions. A robust literature suggests that for behavioral interventions to have long term impact 'booster' sessions are necessary. We will have participants return at 6 and 12 months post HABIT for a one-day booster session where they will first complete all follow-up measures then receive one-hour sessions of the 4 intervention components of their particular study arm.

1) Physical Exercise

Participants will engage in daily 45-60 minutes of physical exercise via yoga. We use yoga as it is suited to the constraints of space and the different levels of baseline physical activity of our participants and partners. HABIT uses an adapted Hatha Yoga practice where participants sit on chairs for some asana (poses) and use the chair for support during the balance and other standing poses and for others parts of the sequence. This adapted Hatha Yoga style is appropriate for older adults and is beneficial and accessible for those who have limited mobility, including those with walkers or who are in wheelchairs. HABIT yoga also incorporates breathing and meditation and cultivates an overall sense of connection and support. Instructors have at least 200 hours of training and are certified. The appropriately sequenced HABIT yoga practice meets the American College of Sports Medicine recommendation for older adults for muscle strengthening and flexibility. The sessions use an armless sturdy chair placed on top of a sticky mat. Standing poses, supported by a chair if needed, increase leg and torso strength, improve the ability to complete activities of daily living and reduce the likelihood of falls. Standing poses support thigh strength, which facilitates ongoing walking and mobility. Balance poses, supported by a chair if needed, also support muscle control to reduce the risk of falling and increases ability to perform activities of daily living. Other yoga poses in the HABIT yoga sequence include those that focus on core strength, joint range of motion, joint stability and overall balance. Physical adjustments or corrections to participant's postures are avoided unless there is a concern with safety. Instructions are mirrored for the participants (the instructor faces the students and does a posture on the left side while instructing the right side to reduce confusion). Breathing practice focuses on increasing lung capacity and oxygenation. The sessions include meditation practice to support internal focus. Specific session structure is listed in the Appendix B.

The HABIT physical exercise intervention is intended to initiate and sustain a schedule rather than a type of physical activity. Post-programming, participants and partners will be encouraged to maintain a schedule of 250 minutes of their preferred exercise per week. Post program we will consider yoga, swimming, walking, running or formal exercise programming (water aerobics, resistance training, etc.) to count equivalently towards this total. Because most clinical

trials of yoga include group classes supported by home practice, HABIT will provide a customized DVD as a supplement for continued use and practice after the program to those that opt to continue yoga. The DVD includes sections on the following: poses, modifications, benefits, breathing, and meditation practices. We will use an activity log completed by the partner to track activity level of the *participants during the 18 month follow-up period.*

2) Computerized brain fitness training

We will use the commercially available Posit Science product BrainHQ™ (www.brainhq.com) on tablets (e.g. iPads). This product is the latest generation of the BrainFitness auditory processing speed program studied by Smith[15] and Zelinski[2] (and now also includes components of the Insight visual processing speed program). Participants will complete 45-60 minutes of training daily in the program and will be encouraged to maintain 250 minutes of computerized brain training per week for *18* months post program. Each participant's adherence and progress will be tracked through the clinician portal provided by Posit Science both during HABIT and for *18* months post-program. Screen shots from the login page and two of the exercises are provided in Appendix C.

3) Wellness Education

The education component is an adaption and synthesis of the Savvy Caregiver psychoeducational program[3] and the "Memory Club" educational program[4]. This education program will involve daily 60-minute group sessions with topics including Introduction to the Program, Living with MCI, Changes in Roles and Relationships, Sleep Hygiene, Steps to Healthy Brain Aging, Preventing Dementia, MCI and Depression, Nutrition and Exercise, Assistive Technologies, and Participating in Research and Community Resources. The slides that serve as the basis for the curriculum are included in the Appendix D.

4) Support Groups

Patient: The patient support group will meet for 45-60 minutes daily. It will use the LifeBio Memory Journal© as a basis for reminiscence-focused group sessions. The table of contents of this product and example pages are reproduced in the Appendix E. Lifebio Memory Journal Page numbers for nightly homework with corresponding themes for the group are also listed in Appendix E.

Partner: The care partner support group meets separately from the patient group for 45-60 minutes daily. It is a traditional support group with no formal curriculum but the following common caregiving themes variously emerge are and addressed in these sessions including: Ambiguity of the Diagnosis, Denial, Disclosure to Friends and Family, Role Changes, Communication, Emotional adjustment, Behavior Changes in our Loved One, Safety, Driving Issues, Planning for the Future, Caregiver Health, Manufacturing Success, Dementia and Relationships, Communication Skills, Defense Mechanisms, Dimensions of Wellness, Effects on Emotions, Family Roles, Grief and Loss, Healthy Relationships, Intimacy Needs, Introduction to Self-Help, Ongoing Care Needs, Spirituality, Stages of Change and Thought Restructuring. Trained group facilitators enhance emotional support, provide guidance about communication approaches, and address denial and the process of grief and loss associated with the diagnosis of MCI in a loved one.

5) Memory Support System (MSS) compensation training.

We will provide each couple with MSS training 5 days per week for 2 weeks, with initial and ending adherence sessions. All sessions will involve 45- 60 minutes of MSS training. The curriculum is described in more detail in Appendix F but described briefly here.

Learning phases. We will utilize three training stages from learning theory[5]: 1) an *acquisition phase* in which use of the MSS is learned, 2) an *application phase* in which a participant is taught to apply MSS use to his/her daily life, and 3) an *adaptation phase* in which a participant practices incorporating the MSS into his/her daily life so as to make its use habitual.

Intervention Plan/Questions. This set of questions used in each session was constructed to help the participants learn each training phase (Also in Appendix F). These questions cover the topics to be learned in each phase of training.

Participants progress to the next training phase after demonstrating 100% accuracy on the Intervention Plan/Questions in a stage for two consecutive days.

Homework. In addition to asking the Intervention Plan/Questions, homework will be given at the end of session to focus on the practice of a MSS skill.

Importance of the care partner. We are aware that even in cognitively intact people, 10 hours of direct training may be insufficient for the acquisition of a new procedural learning skill[6]. As such, we include a care partner in the training to help with cuing and practice outside of the therapy sessions.

Adherence. MSS Adherence (i.e., how well an individual utilizes all sections of their MSS calendar system) will be defined as a score of seven or greater on the Adherence Assessment (Appendix F). The Adherence Assessment will be given on three occasions: 1) on the first day of the intervention, 2) the last day of the intervention, and *6,12-and 18* months post HABIT. The evaluator will examine MSS compliance for two days that are randomly selected from the prior week. Random days are selected to offset the possibility of a participant "preparing" the calendar for their visit.

Outcomes

The table below lists the current outcome measures employed in the clinical HABIT program. As delineated in Aim 1 we will vet these measures with our advisory group in a focus group setting to assess comprehensiveness as well as degree of respondent burden. If the focus groups identify important outcomes we are not measuring we will identify valid measures for those areas and add them to our outcomes assessment. If focus groups identify any existing measure as extraneous and this is confirmed via the survey of all past HABIT participants we will drop that measure.

Treatment Efficacy Measures Proposed to the Patient and Partner Advisory Group (PPAG)

Target:	**ADL**	**Mood**	**Anxiety**	**Self Efficacy (SE)**	**Quality of Life (QOL)**	**Care partner Burden (CB)**
**Participant**	ECog<br>FAQ	CES-D	AIF	SE in MCI*	QOL-AD	
**Care partner**		CES-D	AIF	Caregiver SE	QOL-AD	CB

Note. ADL=Activities of daily living, ECog = Everyday Cognition, FAQ = Functional Assessment Questionnaire, CES-D = Center for Epidemiological Studies Depression scale, QOL-AD = Quality of Life-AD, CB = Caregiver Burden, AIF = Anxiety Inventory Form *Modified from [7], SE=Self Efficacy

Functional Status. ADL functional status ratings based on informant assessment will be obtained at baseline, intervention completion, and *6* months, *12* months *and 18 months post intervention*. The Everyday Cognition (E-Cog; [8] will be used to assess impairments in instrumental activities of daily living (IADL). The E-Cog is an informant-based measure that assesses a participant's ability to perform everyday tasks in the following areas: memory, language, visuospatial abilities, planning, organization, and divided attention. It was constructed specifically to be sensitive to changes in MCI. Factor analysis supports a seven-factor structure including one global factor and six domain-specific factors. The global factor is strongly correlated with Clinical Dementia Rating (CDR) score, MMSE, and clinical diagnosis. In addition to the global factor, the Everyday Memory factor differentiates MCI from normal cognition, and the Everyday Language factor differentiates MCI from dementia. Test-retest reliability over an average of 29 days is good (r = 0.82).[8] The E-Cog was modified with its author's support to assess the participant's *current* functional ability at each time point rather than the original wording comparing functioning to 10 years prior so as to better gauge change from baseline to follow-up. Additionally, in anticipation of future long-term follow-up, ADLs and IADLs will further be assessed with the Functional Assessment Questionnaire (FAQ*) at baseline and 18 months post intervention*. The FAQ is the standard functional measure required for use throughout the Alzheimer's disease Research Centers network. FAQ was developed for use with dementia patients but has also shown to discriminate between normal controls and those with MCI.[9] The FAQ will also be useful in eventual longitudinal follow-up to measure more advanced ADL impairments than may be assessed using the E-Cog.

Mood. At all assessment points, both the subject and the care partner will complete the Center for Epidemiological Studies Depression Scale (CES-D). In addition, both the MCI participant and the care partner will complete the Anxiety Inventory Form (AIF), a 10-item rating scale modified from the State-Trait Anxiety Inventory [10] by the Resources for Enhancing Alzheimer's Caregiver Health (REACH) project.[11]

Quality of Life. Both the participant and care partner will complete the Quality of Life-AD (QOL-AD).[12] The QOL-AD is a 13-item measure developed for individuals with dementia that has been utilized in MCI and with care partners. Participants and care partners rate their relationships, concerns about finances, physical condition, mood, energy level, memory, aspects of daily functioning, and overall life quality on a four-point scale.

Participant Self-efficacy. The MCI participant will complete a measure of self-efficacy at all assessment points using modified, selected items from the Chronic Disease Self-Efficacy Scales.[7] The entire three-item Do Chores Scale, 2-item Social/Recreational Activities Scale, and four items of the 5-item Manage Disease in General Scale were utilized based on their relevance to MCI. Original scales have reported internal consistency reliability of $r \geq .82$ and test-retest reliability of $r \geq .84$.[7] The language from the original scales was modified to be specific to those with MCI (i.e., "your memory/cognitive difficulty" rather than more general references to "your health condition"). The result is the 9-item Self-Efficacy in Mild Cognitive Impairment scale.

Participant Self-compassion. The MCI participant will complete a measure of self-compassion at all assessment points using the 12-item short form of the Self-Compassion Scale[57]. This form has high internal consistency reliability of $r= .87$ and a very high correlation r=.97 with the full 26-item Self-Compassion scale.

Gratitude: The MCI participant will complete 6- item The Gratitude Questionnaire Scale[58] at all assessment points. This Likert scale also has high internal consistency reliability of $r= .82$ and a high fit index (.95) for a single factor model, and good concurrent validity[58].

Caregiver Burden. Care partners will complete the short form of the Caregiver Burden Inventory[13] at all assessment points. This measure is an assessment of degree of stress experienced by family caregivers. It includes 12 questions concerning the effect of the participant's disability on care partners' lives. It is scored as a composite measure, combining several aspects of caregivers' reactions.

Caregiver Self-Efficacy. Care partners will complete the Caregiving Competence and Mastery components of the Pearlin[14] at all assessment points. The measures reflect their titles and range from 4-6 items.

Caregiver Self-Efficacy and Gratitude. Care partners will complete the Short form Self-compassion scale[57] and Gratitude Questionnaire[58] at all assessment points.

Ancillary Data
Clinical Dementia Rating (CDR)[15]. The CDR is an informant based interview designed to stage dementia. The patient is rated on six dimensions; Memory, Orientation, Judgment and Problem solving, Community Affairs, Home and Hobbies, and Personal Care and assigned a Global score is generated via an established algorithm. A Global score of less than or equal to 0.5 is required for enrollment into the study.

Global Cognitive Function. The DRS-2[16] will be given to help determine overall level of cognitive functioning at the initial eligibility session. Although all participants have a diagnosis of amnestic MCI, this measure is given to help determine that the participant has not progressed to dementia since their diagnosis was originally obtained. *This will also be given at the 18 month follow-up point to characterize final global cognitive functioning.*

The MMSE[17] is a widely used screening measure of cognitive impairment. The MMSE will be given to care partners at the eligibility session to determine if global cognitive functioning is intact. A score of greater than 24 is required for enrollment in the study.

The modified version of the Telephone Interview of Cognitive Status-Modified (TIC-M) is similar in scope to the well-known Mini-Mental State Examination (MMSE), but includes a more comprehensive assessment of memory. Subjects who scored within a predefined range met operational criteria for aMCI.

Physical function; Our yoga instructors will administer a timed 400-meter walk and the Short Physical Performance battery which includes a timed 4-M walk, standing side by side, semi-tandem and full tandem stance, and a timed arms-folder rise from seated-to-standingx4.[59]

Enrollment. De-identified records will be kept on all HABIT patients not enrolling in the study, whether this it due to ineligibility or declining consent. Age, education, ethnicity, global cognitive status, treatment success and reason for non-enrollment will be tracked.

Participant Retention. Records will be kept of any subject failing to complete the 10 days of intervention or subsequent follow up assessments.

Timing of Assessment Measures

Measure	Enrollment	Baseline	Intervention Completion	6 Month Booster	*12 Month Booster**	*18 Month Post (mail out)*
**DRS-2**	X (30 min)				*X*	
**MMSE**	X(5 min)					
CDR	*By phone prn*					*By phone*
**Cogstate**		X (20 min)			*X*	
**SPPE**		X (5 min)	X (5 min)		X	
**Calendar Adherence**		X	X	X	*X*	*X* (by mail)
**Participant CES-D**		X	X	X	*X*	*(by mail)*
**Participant QOL**		X	X	X	*X*	*(by mail)*
**Participant SE**		X	X	X	*X*	*(by mail)*
**Participant AIF**		X	X	X	*X*	*(by mail)*
**Participant SC**		X	X	X	*X*	*(by mail)*
**Participant G**		X	X	X	*X*	*(by mail)*
**FAQ**		X	X	X	*X*	*(by mail)*
**CB**		X	X	X	*X*	*(by mail)*
**ECOG**		X	X	X	*X*	*(by mail)*
**Partner CES-D**		X	X	X	*X*	*(by mail)*
**Partner QOL**		X	X	X	*X*	*(by mail)*

**Partner SE**		X	X	X	*X*	*(by mail)*
**Partner AIF**		X	X	X	*X*	*(by mail)*
**Partner SC**		X	X	X	*X*	*(by mail)*
**Partner G**		X	X	X	*X*	*(by mail)*
**Activity log**		X		At random interval	At random interval	

Bring patients in for assessment up to a week before baseline/booster as possible If needed Enrollment DRS, MMSE, and baseline Cogstate and SPPE can be done on same visit including on day 1. A DRS/MMSE/CDR done within 3 months of day 1 can be used for that measurement. Note. Yellow Highlighted measures are to be done blinded study coordinator/psychometrist. Red highlighted measure by MSS therapist. Blue highlighted measures self-administered by patient. Green highlighted measures self-administered by partner. DRS-2=Dementia Rating Scale-2nd Edition, MMSE=Mini Mental State Exam, CDR=Clinical Dementia Rating, FAQ=Functional Assessment Questionnaire, SPPE=Short Physical Performance Examination, SE=Self Efficacy, SC=Self-Compassion, and G=Gratitude, QoL=Quality of Life, CES-D=Depression Questionnaire, CB=Caregiver Burden, AIF=Anxiety

Data management

The study will utilize web-based electronic data capture forms using REDCap software[18] previously created for use in our R01NR012419. Additional forms needed for the proposal will be created in REDCap under the supervision of Dr. Pankratz, our statistician. The forms will be securely accessible at each site from computers or mobile devices with a Web browser. The data forms Website and data files will be stored on a server hosted by the Mayo Clinic CTSA (Grant UL1 RR024150).

REDCap is a secure Web-based application for building and managing online databases for clinical research studies developed within the CTSA consortium. Mayo Clinic is a member of the REDCap Consortium, which is comprised of 372 active institutional partners. REDCap is currently in use for 35,000 studies and had 48,000 users. The REDCap application uses PHP + JavaScript programming languages, and a MySQL database engine. Hardware requirements are modest, and the system runs in Windows and Linux Web server environments.

Users at each site will log in to the data forms with a login ID and password. Individual users will be granted specific user rights determining whether they can view, add, change, lock, or delete records, which site's data they are able to access, and whether summary statistics may be viewed. In addition, all user activity will be documented by an audit trail in a log file. For every data value it will be possible to determine who entered the data, whether it was changed, and what the previous values were. Data export will be limited to the statistician. SAS and/or R will be used for data analysis.

Data analysis

Aim 1 As noted above we will use established techniques[19,20], including data reduction, generation of themes, and validation of themes and findings to draw conclusions from the qualitative data from our PPAG and SAG focus group sessions. We will then obtain descriptive summaries from the surveys that are completed by clinical HABIT alumni couples. We will verify and extend our preliminary outcomes priority data in order to identify recommendations to improve the scope of our outcomes, and/or the nature of our interventions and experimental approach (See patient-centeredness section below). If additional critical outcome variables are identified, we will identify valid measures of those outcomes and add them to the outcomes matrix. Overall efficacy assessment will then be predicated on significant improvement over baseline in the most highly valued outcomes.

Aim 2. Our experience to date is that once enrolled in HABIT there is essentially no treatment dropout. But this is for the full program. Suppression of 1 component in this study might nominally increase treatment dropout but this will still result in *55-60* participants per arm. The more common post-treatment attrition will be death and lost to follow-up post

treatment. To date our attrition rate is less than 10%. We will also conduct sensitivity analysis by conducting a full completers analysis.

In order to determine the degree to which the different components of the HABIT program contribute to improvements in each of the targeted outcomes, we will utilize a linear mixed effect models approach *that accounts for the randomized complete block design that will be employed in the assignment of treatment combinations to study sessions within each site.*. In these models, we will include indicators for the different treatment effects, as well as indicators for potential confounding features such as age, sex, study site, and study session. Using these analyses, we will test for improvements from baseline over follow-up by testing for the significance of interactions between the variables representing study components and those reflecting follow-up period. These tests are similar to paired t-tests but are more flexible. For example, they allow the simultaneous analysis of data from more than two time points, and they enable the inclusion of covariates that may differ from baseline to follow-up.

We will examine the significance of the different treatment effects both with and without adjustment for potential confounders such as gender, age and baseline scores on the Dementia Rating Scale. We will also explore potential heterogeneity of treatment effects by assessing interactions between treatments and gender, age and baseline Dementia Rating Scale score of the patient and relationship of the partner (spouse vs. other).

Aim 3. We also aim to illustrate the efficiency of our novel design and statistical analysis methods for the evaluation of multicomponent interventions. Multicomponent interventions are becoming more common (e.g. anti-retroviral triple therapy for HIV, surgery+radiation+chemotherapy in cancer), and the need for multicomponent studies is so critical that the Food and Drug Administration has gone so far to issue a plan for how to ensure combination (multicomponent) trials meet its standards.[21] Typical clinical trials often contrast one treatment to another (often a placebo). In the case where multiple treatments are studied, it is possible to employ fractional factorial designs.[22] In these designs, subsets of experimental combinations are carefully selected to enable the estimation of treatment effects of interest. As these designs include an experimental condition with no active treatments, and others with only one or two, we opted to pursue a different experimental approach. In this approach, we formed five distinct treatment group combinations by removing a single component of the five possible components comprising the HABIT program. As part of this proposed effort, we will assess the efficiency of this study design in comparison to two-arm studies (i.e. treatment vs. control) and fractional factorial designs. In our application of this study design, we will randomly assign one of these five treatment combinations to study session, stratified by study location in such a way that imbalance is minimized. Our current study design meets the following constraints. (1) Each study site (AZ, FL and MN) is given each candidate treatment combination once. (2) Each treatment combination is given *3* times, for a total of *15* sessions of treatment.

Power analysis

In Aim 2, we will conduct a randomized trial to assess the ability of the different components of the HABIT program to effect changes, and it is this component of the study which requires adequate statistical power. As outlined above, analyses will be based on data gathered from approximately *300 i*ndividuals with amnestic MCI participating in one of a total of *20* sessions. Given the randomized complete block study design employed to assign treatment combinations within study sites, we used approaches developed for this design to estimate the power to conclude that a specific HABIT component provided benefit. Data from our existing clinical sample and a matched untreated control group provided initial estimates for this simulation. We computed the expected variance of the estimated treatment effect for one HABIT component by extracting the appropriate value from the variance-covariance matrix derived from the design matrix corresponding to the allocation of treatment groups within the study. Using this, we estimated the magnitude of the effect size (difference score divided by its standard deviation) that is detectable with 80% power using a two-sided 0.05 level test. The results of this effort suggest that we have 80% power to conclude that a treatment component is efficacious if it is associated with an improvement of 0.53 standard deviation units (d = 0.53) while accounting for effects due to the other treatment components, study sites and sessions within sites. We have observed differences larger than this in previous studies. For instance, we observed that training in the MSS improves E-cog scores by nearly 0.9 standard deviations at

first follow-up in a previous study.[23] Therefore, the proposed study will provide sufficient power to detect the meaningful changes that we expect to observe.

Timeline

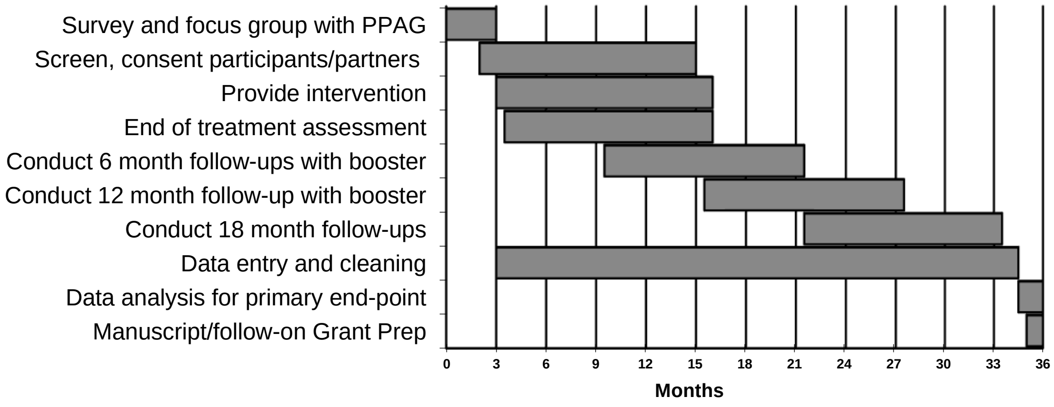

Limitations

This is not a double blinded trial, so the investigator will be aware of the intervention component that was missing and could conceivably bias outcomes. Informed consent will also result in participants' awareness that they are missing one component (e.g., education) possibly impacting their expectations or leading them to identify and initiate that component on their own (e.g., exercise). In an attempt to measure this possibility, we will inquire at follow-up visits about other activities individuals engaged in outside of HABIT recommendations.

Aside from the limitations imposed by the above design considerations, the project has other limitations. For example, this intervention targets participants with amnestic MCI. This is because the intervention targets memory impairments consistent with likely underlying AD pathology and not other cognitive deficits such as language dysfunction. As such, these results may not be generalizable to individuals with non-amnestic MCI subtypes. These populations could certainly be targets for future research.

Also, this grant period runs for only 3 years, limiting the *number of sessions and* length of the follow-up *we can achieve* for these participants.

Finally, we do not believe this intervention will have disease modifying effects and have no mechanism for assessing if it does. The goal of the intervention is to help people with amnestic MCI maintain functional status in spite of probable progression of their disease pathology.

Human Subjects Involvement, Characteristics, and Design. The *300* participants in this study will be men and women with amnestic (single- or multiple-domain) Mild Cognitive Impairment (MCI). Care partners will include spouses, adult children, or a close relative or friend of the person with amnestic MCI, and they will also be providing information about themselves. All persons with amnestic MCI will be over 50 and most will be over 65. The majority of care partners will be spouses also over 50. Adult children or relatives/friends may be younger, but all will be adults over age 18. All patients with a diagnosis of MCI at the Mayo Clinic and the University of Washington memory disorders clinic will be provided a recommendation to participate in the HABIT program. Consecutive HABIT referrals meeting the Inclusion/Exclusion Criteria (see Research Plan) will be contacted via telephone about participation. Subjects will not be excluded based on medical status. As this is a geriatric sample in which the women are postmenopausal, no pregnant women or women of childbearing potential are included. If any care partners are younger women of childbearing potential, this study poses no additional risk to them.

Three Mayo Clinic sites in Rochester, Minnesota, Scottsdale, Arizona and Jacksonville, Florida and University of Washington Harborview Medical Center will participate in recruiting human subjects in this proposed study. Rochester

will serve as both the Training and Data Core. Data will be obtained from subjects via clinical records, and interaction with HABIT study staff. The proposed study will utilize web-based electronic data capture forms using REDCap software[48] previously created for use in our R01-NR012419 and stored on a server hosted by the Mayo Clinic CTSA (Grant UL1 RR024150). REDCap is a secure Web-based application for building and managing online databases for clinical research studies. Mayo Clinic is a member of the REDCap Consortium, which is comprised of 372 active institutional partners. REDCap is currently in use for 35,000 studies and had 48,000 users. Information in REDCap will be scrubbed of Protected Health Information and utilize research IDs.

Sources of Material. Data will be collected specifically for the conduct of the proposed research. We will collect information concerning demographics such as age and education, medical history, current medications. Participants and partners will complete outcome measures including Memory Support System (MSS) Adherence, mood, quality of life, self-efficacy, and measures of the patients' functioning, including basic activities and instrumental activities of daily living in sessions lasting approximately 90 minutes. All data obtained in this study will remain confidential. The identification key to each research ID will be maintained under lock and key at each participating site. Database information will be password protected, and hardcopies of patient information will be kept in a locked cabinet at each site. Only the PIs, MSS trainers, education leaders, and psychometricians specific to each site will have access to patient identities.

Potential Risks. The most common risks and discomforts expected in this study are:

- Fatigue from completing the evaluation.
- Anxiety about cognitive challenges
- Stress from facing the diagnosis of MCI/Alzheimer's disease
- Threats to confidentiality due to group nature of the intervention.

Adequacy of Protection Against Risks

Recruitment and Informed Consent. Potential subjects will be identified by the program coordinator or one of the investigators on this project via HABIT referrals at each site. Potential participants and their legal proxies, usually their primary care partner, will be called via telephone notifying them they may qualify for this project. We will also utilize advertising via brochures, flyers, newspaper articles, or other means suggested through the Mayo Clinic and ADRC as appropriate.

Informed consent may be obtained by the principal or co-investigators or program coordinator prior to or at the beginning of the initial visit. Given subjects' mildly impaired status without dementia, they should be able to provide informed consent. All care partners will also provide consent.

Protection Against Risk. To reduce the risk, including loss of confidentiality, a code will identify all participants, and all data will be maintained in a secure file cabinet. Computerized data files will identify the participant by study number rather than name or other universal identifier. Files that associate names and study numbers will be kept in a locked file in hard copy format. Trained psychometrists will collect psychometric data. The neuropsychologist site PI will be available during the testing of patients and the MSS training should adverse events arise. Persons may choose to withdraw from this study without prejudice to their subsequent care at each site or their involvement in the ADRC. Participants will be given a copy of the consent listing the PIs' contact information.

Potential Benefits of the Proposed Research to the Subjects and Others

The proposed research will help us understand which interventions provide the most benefit for which outcomes from each HABIT intervention component. Tailoring behavioral interventions to the most valued outcomes for each individual will help improve the lives of individuals with MCI and their care partners. Behavioral interventions in MCI may delay onset of dementia in those with progressive illness, and this may also lead to successful prevention models for AD.

The risks to the participants are reasonable given the significant amount of resources that can be saved in the future when providing multiple hours of behavioral interventions tailored patients' values and priorities.

Importance of the Knowledge to be Gained

Individuals with MCI and their care partners often want to learn ways to manage and understand the memory loss they are experiencing. Frequently, the memory problems experienced by individuals with amnestic MCI negatively impact their daily lives, including mood, family relationships, general health/treatment compliance, and eventual independent living status. This research project will help us gain more knowledge as to which patients may best benefit from behavioral interventions, in particular the cognitive and physical exercises, support groups and memory compensation training intervention. The potential knowledge to be gained outweighs the anticipated risks.

Data and Safety Monitoring Plan

The PI at each site will be responsible for monitoring the safety and efficacy of this trial and executing the Data and Safety Monitoring Plan (DSMP). In this study, we will use the FDA definition of serious adverse events (SAEs) and adverse events (AEs). SAEs are unlikely in a behavioral intervention. Any SAE, whether or not it is related to the study intervention, will be reported to the site IRB. The PI at each site will monitor for the presence of both SAEs and AEs at each scheduled visit. These individuals will report any SAE or AE to the overall PI twice a month. However, if the SAE involves death or a life-threatening event, the site PI will be notified within 24 hours, and the Principal Investigator will notify the IRB within 2 working days from the time the SAE was first reported. Reports of SAEs received by the IRB will be reviewed by an institutional SAE board to make a determination of the seriousness of the event and to determine what actions, if any, will be required.

In the event that a participant withdraws from the study or the PI decides to discontinue a participant due to an SAE, the participant will be monitored by the site PI via ongoing status assessment until 1) resolution of the problem is reached or 2) the SAE is determined to be clearly unrelated to the study intervention. The DSMP will be reviewed on a monthly basis by the PI and other co-investigators as necessary. Summary and outcome of all SAEs will be reported to the IRB annually.

REFERENCES CITED

1. Albert M, DeKosky S, Dickson D, et al. The diagnosis of mild cognitive impairment due to Alzheimer's disease: recommendations from the National Institute on Aging-Alzheimer's Association workgroups on diagnostic guidelines for Alzheimer's disease. Alzheimer's & Dementia : The Journal of the Alzheimer's Association 2011;7:270-9.
2. Zelinski EM, Spina LM, Yaffe K, et al. Improvement in memory with plasticity-based adaptive cognitive training: results of the 3-month follow-up. Journal of the American Geriatrics Society 2011;59:258-65.
3. Hepburn K, Lewis M, Tornatore J, Sherman CW, Bremer KL. The Savvy Caregiver program: the demonstrated effectiveness of a transportable dementia caregiver psychoeducation program. Journal of gerontological nursing 2007;33:30-6.
4. Gaugler JE, Gallagher-Winker K, Kehrberg K, et al. The Memory Club: Providing support to persons with early-stage dementia and their care partners. Am J Alzheimers Dis Other Demen 2011;26:218-26.
5. Sohlberg MM, Mateer CA. Training use of compensatory memory books: a three stage behavioral approach. Journal of clinical and experimental neuropsychology 1989;11:871-91.
6. Lally P, van Jaarsveld CHM, Potts HWW, Wardle J. How are habits formed: Modelling habit formation in the real world. European Journal of Social Psychology 2010;40:998-1009.
7. Lorig K, Stewart, A, Ritter, P, Gonzalez, V, Laurent, D. & Lynch, J. Outcome Measures for Health Education and other Health Care Interventions. Thousand Oaks (CA): Sage Publications; 1996.
8. Farias ST, Mungas D, Reed BR, et al. The measurement of everyday cognition (ECog): scale development and psychometric properties. Neuropsychology 2008;22:531-44.
9. Teng E, Becker BW, Woo E, Knopman DS, Cummings JL, Lu PH. Utility of the Functional Activities Questionnaire for Distinguishing Mild Cognitive Impairment From Very Mild Alzheimer Disease. Alzheimer Dis Assoc Disord 2010.
10. Spielberger CD, Gorssuch, R.L., Lushene, P.R., Vagg, P.R., Jacobs, G.A. Manual for the State-Trait Anxiety Inventory. : Consulting Psychologists Press, Inc; 1983.
11. Wisniewski SR, Belle SH, Coon DW, et al. The Resources for Enhancing Alzheimer's Caregiver Health (REACH): project design and baseline characteristics. Psychol Aging 2003;18:375-84.
12. Logsdon RG, Gibbons LE, McCurry SM, Teri L. Assessing quality of life in older adults with cognitive impairment. Psychosom Med 2002;64:510-9.
13. Bédard M, Molloy DW, Squire L, Dubois S, Lever JA, O'Donnell M. The Zarit Burden Interview: A New Short Version and Screening Version. The Gerontologist 2001;41:652-7.
14. Pearlin LI, Mullan JT, Semple SJ, Skaff MM. Caregiving and the stress process: an overview of concepts and their measures. Gerontologist 1990;30:583-94.
15. Morris J. The Clinical Dementia Rating (CDR): current version and scoring rules. Neurology 1993;43:2412-4.
16. Jurica PJ, Leitten, C., Mattis, S Dementia Rating Scale-2; Professional Manual. Lutz, FL Psychological Assessment Resources, Inc; 2004.
17. Folstein M, Folstein S, McHugh P. "Mini-mental state." A practical method for grading the cognitive state of patients for the clinician. Journal of Psychiatry Research 1975;12:189-98.

18. Harris PA, Taylor R, Thielke R, Payne J, Gonzalez N, Conde JG. Research electronic data capture (REDCap)--a metadata-driven methodology and workflow process for providing translational research informatics support. J Biomed Inform 2009;42:377-81.
19. Devers KJ. How Will We Know "Good" Qualitative Research When We See It? Health Services Research 1999;34 S1153 88.
20. Miles MB, Huberman AM. Qualitative data analysis: An expanded sourcebook (2nd ed.). Thousand Oaks, CA Sage; (1994).
21. Services USDoHaH. Guidance for Industry: Codevelopment of Two or More New Investigational Drugs for Use in Combination. In: Administration FaD, ed. Washington D.C.: U.S. Government; 2013.
22. Nair V, Strecher V, Fagerlin A, et al. Screening experiments and the use of fractional factorial designs in behavioral intervention research. Am J Public Health 2008;98:1354-9.
23. Greenaway MC, Duncan NL, Smith GE. The memory support system for mild cognitive impairment: randomized trial of a cognitive rehabilitation intervention. Int J Geriatr Psychiatry 2013;28:402-9.
