# Supplementary material for: Comparative Effectiveness of Behavioral Interventions on Quality of Life for Older Adults With Mild Cognitive Impairment: A Randomized Clinical Trial
Source: JAMA Netw Open. 2019 May 17;2(5):e193016. doi: 10.1001/jamanetworkopen.2019.3016 (PMC6537922; doi:10.1001/jamanetworkopen.2019.3016)
Supplement: Supplement 2. — eFigure. Mean Changes in Quality of Life eTable 1. Baseline Characteristics According to Length of Follow-up eTable 2. Outcome Measures at Baseline by Study Group and Site eTable 3. Summaries of Raw Changes in Outcome Measures from Baseline to 12 Months by Study Group and Site [file jamanetwopen-2-e193016-s002.pdf]

# Supplementary Online Content

Chandler MJ, Locke DE, Crook JE, et al. Comparative effectiveness of behavioral interventions on quality of life for older adults with mild cognitive impairment: a randomized clinical trial. *JAMA Netw Open*. 2019;2(5):e193016. doi:10.1001/jamanetworkopen.2019.3016

**eFigure.** Mean Changes in Quality of Life

**eTable 1.** Baseline Characteristics According to Length of Follow-up

**eTable 2.** Outcome Measures at Baseline by Study Group and Site

**eTable 3.** Summaries of Raw Changes in Outcome Measures from Baseline to 12 Months by

Study Group and Site

**This supplementary material has been provided by the authors to give readers additional information about their work.**

© 2019 Chandler MJ et al. *JAMA Network Open*.

eFigure. Mean Changes in Quality of Life

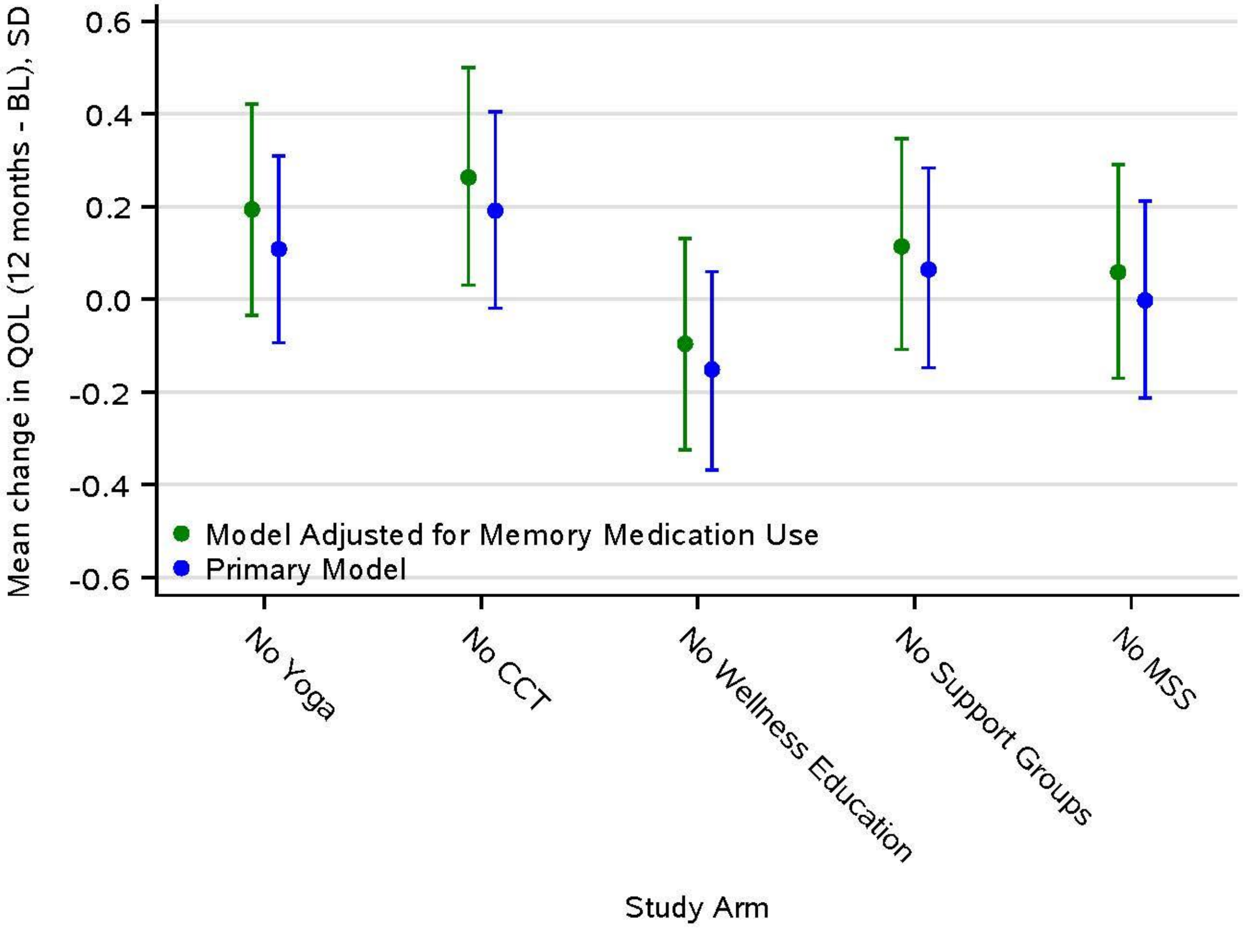


© 2019 Chandler MJ et al. *JAMA Network Open*.

**eTable 1.** Baseline Characteristics According to Length of Follow-up

| Characteristic | Less than 12 months follow-up (n=44) | 12-18 months follow-up (n=228) |
|---|---|---|
| Age, years | n=44 | n=228 |
| Mean (SD) | 77.4 (7.7) | 74.7 (7.5) |
| Sex, No (%) | n=44 | n=228 |
| Male | 22 (50) | 138 (61) |
| Female | 22 (50) | 90 (39) |
| Years of education | n=44 | n=227 |
| Median (range) | 16 (12-20) | 16 (6-20) |
| Race, No (%) | n=44 | n=228 |
| Non-white | 2 (5) | 10 (4) |
| White, non-Hispanic | 42 (95) | 218 (96) |
| Marital status, No (%) | n=40 | n=228 |
| Married or marriage-like relationship | 31 (78) | 204 (89) |
| Divorced or separated | 2 (5) | 7 (3) |
| Widowed | 6 (15) | 14 (6) |
| Single and never married | 1 (3) | 3 (1) |
| Caregiver relationship to patient, No (%) | n=40 | n=228 |
| Spouse or partner | 30 (75) | 199 (87) |
| Son or daughter | 8 (20) | 15 (7) |
| Brother or sister | 0 (0) | 5 (2) |
| Other family member | 0 (0) | 2 (1) |
| Friend | 2 (5) | 6 (3) |
| Other | 0 (0) | 1 (0) |
| Current memory medications, No (%) | n=46 | n=228 |
| No | 23 (64) | 141 (62) |
| Yes | 13 (36) | 87 (38) |
| Dementia Rating Scale-2 total score | n=41 | n=221 |
| Mean (SD) | 127.8 (7.6) | 129.5 (8.7) |
| Clinical Dementia Rating scale score, No (%) | n=44 | n=227 |
| 0=None | 6 (14) | 15 (7) |
| 0.5=Questionable | 38 (86) | 212 (93) |
| Anxiety score | n=36 | n=226 |
| Mean (SD) | 17.0 (5.7) | 17.8 (5.2) |
| QOL- score | n=37 | n=227 |
| Mean (SD) | 40.0 (5.2) | 40.4 (5.7) |
| Mood score | n=37 | n=226 |
| Mean (SD) | 12.8 (6.9) | 11.8 (8.3) |
| Self-efficacy score | n=36 | n=227 |
| Mean (SD) | 73.8 (13.9) | 73.9 (14.0) |
| mADL score | n=36 | n=227 |
| Mean (SD) | 19.1 (5.6) | 18.6 (4.8) |
| | | |

© 2019 Chandler MJ et al. *JAMA Network Open*.

Abbreviations: No, number; SD, standard deviation; QOL, Quality of Life; mADL, memory-related activities of daily living.

© 2019 Chandler MJ et al. *JAMA Network Open*.

**eTable 2.** Outcome Measures at Baseline by Study Group and Site

| Outcome | Site | **No Yoga** | | **No CCT** | | **No Wellness Education** | | **No Support Groups** | | **No MSS** | |
|---|---|---|---|---|---|---|---|---|---|---|---|
| | | N | Mean (SD) | N | Mean (SD) | N | Mean (SD) | N | Mean (SD) | N | Mean (SD) |
| | | | | | | | | | | | |
| QOL | MN | 22 | 41.7 (4.8) | 12 | 39.2 (6.1) | 18 | 39.1 (4.7) | 17 | 41.2 (5.2) | 19 | 40.5 (4.8) |
| | FL | 13 | 40.6 (5.3) | 9 | 40.0 (2.6) | 8 | 40.3 (4.0) | 13 | 43.2 (5.1) | 10 | 38.3 (5.2) |
| | AZ | 14 | 35.8 (6.8) | 20 | 42.3 (5.7) | 15 | 39.0 (6.2) | 15 | 41.0 (4.3) | 14 | 40.9 (5.0) |
| | WA | 7 | 41.6 (5.7) | 12 | 41.0 (6.8) | 8 | 38.4 (4.6) | 8 | 40.4 (9.5) | 10 | 38.9 (7.5) |
| | | | | | | | | | | | |
| Mood | MN | 22 | 11.1 (8.9) | 12 | 16.3 (14.7) | 18 | 13.2 (7.3) | 17 | 9.2 (5.3) | 19 | 12.4 (6.4) |
| | FL | 13 | 9.5 (5.7) | 9 | 14.6 (6.0) | 8 | 8.3 (4.7) | 13 | 10.1 (9.0) | 10 | 15.3 (5.7) |
| | AZ | 13 | 14.1 (7.2) | 20 | 9.4 (5.8) | 15 | 12.9 (10.2) | 15 | 11.9 (7.3) | 14 | 10.8 (4.4) |
| | WA | 7 | 11.6 (11.8) | 12 | 11.9 (10.0) | 8 | 15.2 (10.3) | 8 | 11.0 (8.2) | 10 | 14.0 (10.5) |
| | | | | | | | | | | | |
| Self-Efficacy | MN | 22 | 76.5 (13.7) | 12 | 66.0 (22.1) | 18 | 75.8 (9.2) | 17 | 74.5 (14.6) | 19 | 72.7 (16.0) |
| | FL | 13 | 73.8 (10.3) | 9 | 79.1 (9.7) | 8 | 73.3 (10.1) | 13 | 75.1 (16.6) | 10 | 72.8 (14.6) |
| | AZ | 14 | 70.3 (14.8) | 20 | 77.5 (12.9) | 15 | 70.7 (15.4) | 14 | 77.8 (6.3) | 14 | 75.5 (13.0) |
| | WA | 7 | 74.4 (12.5) | 12 | 72.8 (14.7) | 8 | 70.8 (13.3) | 8 | 74.9 (18.6) | 10 | 68.0 (18.1) |
| | | | | | | | | | | | |
| mADLs | MN | 22 | 18.9 (4.9) | 12 | 19.2 (4.0) | 17 | 18.8 (5.4) | 17 | 18.6 (4.3) | 19 | 17.2 (4.4) |
| | FL | 13 | 17.4 (4.0) | 9 | 22.0 (6.5) | 8 | 18.0 (3.5) | 13 | 18.7 (4.7) | 10 | 19.8 (2.6) |
| | AZ | 14 | 17.8 (5.5) | 21 | 18.9 (5.9) | 15 | 16.5 (5.1) | 15 | 18.4 (5.4) | 14 | 20.9 (4.3) |
| | WA | 7 | 18.4 (5.9) | 12 | 17.5 (4.5) | 8 | 21.0 (3.9) | 8 | 20.6 (5.8) | 10 | 18.6 (6.5) |

Abbreviations: CCT, computerized cognitive training; MSS, memory support system; SD, standard deviation; QOL, Quality of Life; mADL, memory-related activities of daily living.

© 2019 Chandler MJ et al. *JAMA Network Open*.

**eTable 3.** Summaries of Raw Changes in Outcome Measures from Baseline to 12 Months by Study Group and Site

| Outcome | Site | **No Yoga** | | **No CCT** | | **No Wellness Education** | | **No Support Groups** | | **No MSS** | |
|---|---|---|---|---|---|---|---|---|---|---|---|
| | | N | Mean (SD) | N | Mean (SD) | N | Mean (SD) | N | Mean (SD) | N | Mean (SD) |
| | | | | | | | | | | | |
| QOL | MN | 18 | -0.2 (3.4) | 11 | 0.9 (2.4) | 16 | 0.5 (4.7) | 15 | 0.2 (4.8) | 16 | -0.5 (3.4) |
| | FL | 12 | -0.3 (4.5) | 5 | 3.0 (3.4) | 7 | -1.1 (3.7) | 8 | -0.6 (2.4) | 8 | 3.0 (3.5) |
| | AZ | 11 | 3.4 (3.5) | 15 | -0.3 (3.6) | 12 | -0.5 (3.5) | 12 | 1.0 (3.7) | 11 | -1.9 (3.7) |
| | WA | 7 | 2.3 (3.7) | 12 | 0.8 (5.5) | 8 | -1.7 (7.1) | 7 | -0.6 (3.6) | 8 | 1.2 (3.7) |
| | | | | | | | | | | | |
| Mood | MN | 18 | -0.4 (6.9) | 11 | -5.5 (11.2) | 16 | 0.6 (5.3) | 15 | 2.5 (6.2) | 16 | 1.9 (5.1) |
| | FL | 12 | 2.8 (6.9) | 5 | -1.4 (3.8) | 7 | 5.2 (6.3) | 7 | -2.2 (4.5) | 8 | -1.9 (7.8) |
| | AZ | 10 | 2.8 (7.3) | 15 | -0.6 (5.6) | 12 | 1.8 (9.2) | 12 | -3.4 (7.1) | 11 | 1.6 (7.4) |
| | WA | 7 | -2.3 (11.6) | 12 | -0.1 (7.1) | 8 | 2.7 (8.2) | 7 | 0.6 (5.0) | 8 | -1.5 (5.0) |
| | | | | | | | | | | | |
| Self-Efficacy | MN | 18 | 2.7 (10.7) | 11 | 2.3 (11.5) | 16 | 1.0 (8.3) | 15 | -7.3 (13.0) | 16 | 3.7 (9.1) |
| | FL | 12 | -6.9 (16.5) | 5 | -0.8 (5.4) | 7 | -1.5 (11.2) | 8 | 0.8 (8.3) | 8 | 3.5 (8.9) |
| | AZ | 11 | 0.9 (5.1) | 15 | -0.2 (8.0) | 12 | 0.4 (9.8) | 12 | -1.3 (10.9) | 11 | -2.2 (14.5) |
| | WA | 7 | -4.6 (15.6) | 12 | 4.1 (12.6) | 8 | -6.6 (20.5) | 7 | -2.1 (17.7) | 8 | 0.6 (12.3) |
| | | | | | | | | | | | |
| mADLs | MN | 18 | 2.1 (4.7) | 11 | -0.1 (4.5) | 15 | 1.2 (4.6) | 15 | 1.7 (3.1) | 16 | 2.3 (4.5) |
| | FL | 12 | 3.1 (5.2) | 5 | -2.4 (4.4) | 7 | 2.3 (3.9) | 8 | -0.5 (0.9) | 8 | 1.8 (4.1) |
| | AZ | 11 | 2.5 (3.2) | 16 | 1.6 (5.3) | 12 | 0.8 (4.8) | 11 | 0.1 (3.1) | 11 | 0.8 (2.9) |
| | WA | 7 | 2.1 (1.9) | 11 | 2.3 (2.5) | 8 | 1.9 (4.8) | 7 | -1.7 (3.7) | 8 | -1.3 (7.8) |

Abbreviations: CCT, computerized cognitive training; MSS, memory support system; SD, standard deviation; QOL, Quality of Life; mADL, memory-related activities of daily living.

© 2019 Chandler MJ et al. *JAMA Network Open*.
