# Supplementary material for: Comparative Effectiveness of Behavioral Interventions on Quality of Life for Older Adults With Mild Cognitive Impairment: A Randomized Clinical Trial
Source: JAMA Netw Open. 2019 May 17;2(5):e193016. doi: 10.1001/jamanetworkopen.2019.3016 (PMC6537922; doi:10.1001/jamanetworkopen.2019.3016)
Supplement: Supplement 3. — Data Sharing Statement [file jamanetwopen-2-e193016-s003.pdf]

Data Sharing Statement

Chandler. Comparative Effectiveness of Behavioral Interventions on Quality of Life for Older Adults With Mild Cognitive Impairment. *JAMA Netw Open*. Published May 17, 2019.
10.1001/jamanetworkopen.2019.3016

Data
Data available: Yes
Data types: Deidentified participant data, Data dictionary
How to access data: The mechanism of data sharing will entail written request for data to be reviewed by the principal investigators for approval (chandler.melanie@mayo.edu). The de-identified data will then be uploaded to an electronic file (spreadsheet or text file) and transferred to the requestor.
When available: With publication

Supporting Documents
Document types: None

Additional Information
Who can access the data: All outside requests for data sharing will need to be approved by our institutional review boards and governed by a Data Use Agreement, an agreement between our institutions and the recipient
Types of analyses: The criteria for providing access to data will include the scientific merit of the proposed work as described in the application and the ability of the investigators to successfully carry out the proposed work.
Mechanisms of data availability: The de-identified data will then be uploaded to an electronic file (spreadsheet or text file) and transferred to the requestor. Study personnel will be available for questions. It is expected that most requests will involve questions, as the data are complex and most work will involve analysis of repeated measurements.
